# A 3D Carbon Architecture Encapsulation Strategy for Boosting the Performance of Nickel Disulfide as an Anode for Sodium-Ion Batteries

**DOI:** 10.3390/molecules29245906

**Published:** 2024-12-14

**Authors:** Yuzhu Li, Mengyuan Zhang, Boying Zhang, Bingke Li

**Affiliations:** 1 College of Chemistry and Pharmaceutical Engineering, Nanyang Normal University, Nanyang 473061, China; 2 School of Biological and Chemical Engineering, Nanyang Institute of Technology, Nanyang 473004, China

**Keywords:** nickel disulfide, sodium storage, 3D carbon encapsulation, high reaction kinetics

## Abstract

Nickel disulfide (NiS_2_) nanoparticles are encapsulated within nitrogen and sulfur co-doped carbon nanosheets, which are grown onto carbon nanofibers to form an array structure (NiS_2_/C@CNF), resulting in a self-supporting film. This encapsulated structure not only prevents the agglomeration of NiS_2_ nanoparticles, but also memorably buffers its volume changes during charge/discharge cycles, thereby maintaining structural integrity. The nitrogen and sulfur co-doping enhances electronic conductivity and facilitates the faster ion transport of the carbon backbone, improving the low conductivity of the NiS_2_/C@CNF anodes. Consequently, the NiS_2_/C@CNF electrode exhibits a remarkable rate ability, reaching 55.4% of its capacity at 5 A g^−1^ compared to that at 0.1 A g^−1^, alongside an impressive cycling stability, with 89.9% capacity retention over 1500 cycles at 2 A g^−1^. This work underscores the efficacy of the 3D carbon backbone encapsulation strategy for enhancing the sodium storage property of transition metal-based anodes.

## 1. Introduction

Lithium-ion batteries (LIBs) have become integral to our daily lives due to their environmentally friendly nature, high energy density, and long cycle life [1,2]. Despite these advantages, the finite and unevenly distributed lithium resources pose enormous challenges to the continuable application of LIBs [3,4]. Given the abundant availability and low cost of sodium resources, sodium-ion batteries (SIBs) have emerged as a strong alternative to LIBs [5,6]. Graphite anodes have been successfully commercialized in LIBs. However, thermodynamic limitations hinder the sufficient intercalation of sodium ions into graphite, making it unsuitable for a direct application in sodium-ion batteries [7]. Moreover, for many of the anode materials used in LIBs, the relatively larger ionic radius and diminished reactivity of sodium ions noticeably compromises their performance in SIBs [8,9]. Thus, the creation of an excellent anode is critical for driving the commercial implementation of SIBs.

Extensive efforts have been made by researchers to explore and enhance the properties of various Na-ion storage materials, such as carbonaceous materials [10,11,12], transition-metal selenides/sulfides/oxides [5,13,14,15,16], and alloys [17]. Among these, nickel disulfide (NiS_2_) has garnered considerable interest in term of its high practical capacity and eco-friendly nature [18,19,20]. Nevertheless, the conversion reactions in NiS_2_ electrodes are accompanied by pronounced volumetric alterations during cycling, resulting in poor cycling stability. Additionally, the inherent low conductivity of NiS_2_ further limits its electrochemical activity [21,22]. To address these weakness, researchers have adopted multiple modification strategies and achieved substantial advancements. These strategies have included structural engineering to create nanoscale morphologies [20,23], heteroatom doping to modify its electronic structure [24], the designing of heterostructures to improve ion transport and electron mobility [21], and combining active materials with conductive carbon matrices to enhance mechanical stability and electrical conductivity [25,26,27]. Carbon composites, specifically, have proven to be highly effective at stabilizing the structure and augmenting the electrochemical behavior of nickel sulfide. For example, Du et al. [28] demonstrated that embedding NiS_2_ particles within carbon fibers effectively prevented the aggregation of nanoparticles. This strategic design, paired with the growth of NiS_2_ nanoflowers on the carbon fiber surface, significantly increased the active surface area available for sodium-ion adsorption, thereby improving the sodium storage performance. This architecture enabled the electrode to deliver an augmented capacitance of 1691.1 F g^−1^ at 1 A g^−1^. Moreover, Xia et al. [23] developed a structurally robust composite electrode by embedding core–shell NiS_2_ particles into carbon fibers, achieving a design that combined excellent mechanical flexibility with enhanced stability. The 3D conductive carbon network facilitated rapid electron transport, while the embedded architecture effectively reinforced the structural integrity of the electrode under prolonged cycling conditions. The resulting NiS_2_/PCF electrode exhibited an outstanding electrochemical performance, retaining 76% of its initial capacity after 5000 cycles at 5 A g^−1^. The sodium storage behavior of carbon materials is crucial when combined with NiS_2_. However, pristine carbon materials often exhibit a suboptimal sodium storage capacity [29]. Heteroatom doping has emerged as an effective strategy with which to optimize the properties of carbon [30,31,32]. For example, N-doping can provide additional active sites for sodium-ion storage [32]. Ma et al. [33] synthesized 2D carbon materials with a tunable carbon layer thickness. The incorporation of nitrogen doping led to a porous carbon characterized by a high specific surface area and an abundant presence of pyridinic nitrogen. These features collectively enhanced the sodium-ion transport and storage capabilities, while optimizing the adsorption and desorption behavior of the charge carriers. Moreover, Wang et al. [34] synthesized N/S co-doped porous carbon, which demonstrated significantly improved conductivity owing to the synergistic effects of the dual doping. The incorporation of N/S not only fortified the carbon matrix with abundant sodium-ion adsorption sites, but also reduced the diffusion barriers for Na^+^. The resulting DF-N/S electrode attained a notable capacity of 327.04 mA h g^−1^ at 0.05 A g^−1^, showcasing the efficacy of the heteroatom doping strategy. Further advancements have been made by Liu et al. [27], who encapsulated nickel sulfide in a N/S co-doped carbon. This design provided dual benefits: it buffered the volumetric changes in nickel sulfide during cycling, thereby stabilizing the electrode structure, and it enhanced the overall electrical conductivity, facilitating efficient charge transport. The encapsulated structure also possessed an elevated specific surface area, bolstering the pseudocapacitive sodium storage. Accordingly, the MNS@NSC electrode exhibited a notable rate property, achieving a specific capacity 569.8 mA h g^−1^ at 5 A g^−1^.

In this work, we boost the reaction dynamics and cycling stability of NiS_2_ nanoparticles via encapsulating them in a three-dimensional (3D) carbon architecture (NiS_2_/C@CNF). This 3D carbon architecture consists of nitrogen and sulfur co-doped carbon nanosheets (NS-CNS) grown onto carbon nanofibers (CNFs). The CNFs act as a robust structural backbone, which not only prevent the agglomeration of NiS_2_ nanoparticles, but also provide continuous electron transport pathways. The NS-CNS, forming an encapsulating layer around the NiS_2_ nanoparticles, create an ample buffer space that accommodates the volumetric change in NiS_2_ during cycling. This encapsulation strategy also enhances the electrical conductivity, ensuring an efficient charge transfer throughout the electrode. The encapsulated structure demonstrates an exceptional cycling capability, retaining a reversible capacity of 513.8 mA h g^−1^ after 150 cycles at 0.2 A g^−1^. Moreover, the electrode showcases impressive high-rate properties, providing a reversible capacity of 321.3 mA h g^−1^ at 5 A g^−1^.

## 2. Results and Discussion

**Figure 1:**
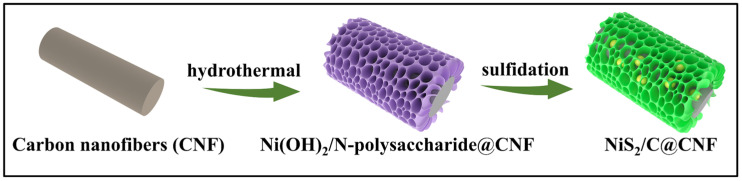
Synthetic illustration of NiS_2_/C@CNF.

**Figure 2:**
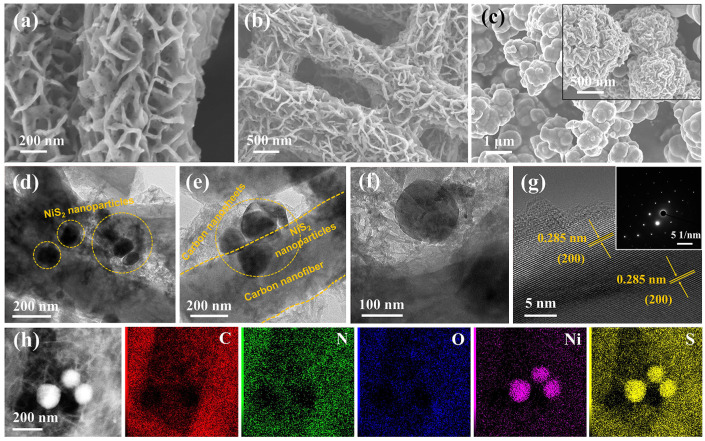
(**a**,**b**) SEM images of NiS_2_/C@CNF. (**c**) SEM images of NiS_2_/C. (**d**,**e**) TEM images of NiS_2_/C@CNF. (**f**) HRTEM and (**g**) selected area electron diffraction of NiS_2_/C@CNF. (**h**) HAADF-STEM image and EDS mappings of NiS_2_/C@CNF.

Figure 1 reveals the production procedure for the NiS_2_/C@CNF. First, carbon nanofibers are produced via electrospinning as well as a subsequent annealing process. Then, Ni(OH)_2_ is embedded into N-polysaccharide nanosheets, which were grown on carbon nanofibers (Ni(OH)_2_/N-polysaccharide@CNFs) using a hydrothermal method. Finally, the Ni(OH)_2_/N-polysaccharide@CNFs undergo vapor-phase sulfidation, during which the N-polysaccharide is transformed into S, N co-doping carbon nanosheets; meanwhile, the Ni(OH)_2_ is converted into NiS_2_ nanoparticles embedded in the carbon nanosheets. Figure 2a,b show the SEM images of the NiS_2_/C@CNF, revealing that its carbon nanosheets are anchored on the carbon nanofiber surface, forming an array structure. The interconnected carbon nanofibers establish a 3D carbon architecture that facilitates rapid electron transport. The NiS_2_/C@CNF film can serve as a self-supporting electrode, eliminating the need for binders. Figure 2c displays the SEM images of the NiS_2_/C, revealing that the carbon nanosheets are densely aggregated into a quasi-spherical morphology, evidently reducing the exposure of the active sites compared to the NiS_2_/C@CNF. Figure 2d,e depict the TEM images of the NiS_2_/C@CNF, indicating that the embedded NiS_2_ nanoparticles have an approximate size of 150 nm within the carbon nanosheets. Notably, the NiS_2_ nanoparticles are not deposited on the carbon fiber surface, but are distributed within the carbon nanosheets, forming an array structure. In this configuration, the carbon nanosheets enhance the conductivity of the NiS_2_/C@CNF sample, thereby improving the electrochemical activity of the sodium ions in the electrode. It is noteworthy that our approach confines the NiS_2_ nanoparticles within the space enclosed by the carbon nanosheets and carbon fibers. This unique architecture offers buffering space for the volumetric change in and prevents the clustering of NiS_2_ during repeated charge–discharge cycles, consequently enhancing the cycling stability and maintaining the exposure of the active sites. As depicted in Figure 2f, the lattice fringe spacing of 0.285 nm relates to the (200) crystallographic plane of cubic-phase NiS_2_, underscoring the well-defined crystalline nature of the NiS_2_/C@CNF. The SAED pattern in Figure 2g further corroborates the single-crystalline structure of the NiS_2_/C@CNF, displaying distinct diffraction spots indicative of high crystallinity and uniformity. Figure 2h displays the distribution of elements C, N, O, Ni, and S within the NiS_2_/C@CNF sample. The specific surface area of the NiS_2_/C@CNF is 25.8 m^2^ g^−1^, which was investigated through nitrogen adsorption–desorption isotherms, as illustrated in Figure S1.

**Figure 3:**
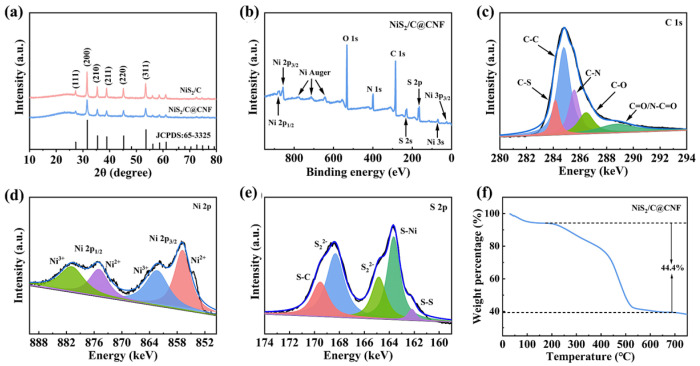
(**a**) XRD patterns of NiS_2_/C and NiS_2_/C@CNF samples. (**b**) XPS survey spectra of NiS_2_/C@CNF. High-resolution XPS spectra of (**c**) C 1s, (**d**) Ni 2p, and (**e**) S 2p of NiS_2_/C@CNF. (**f**) TG curves of NiS_2_/C@CNF.

The crystal structures of the NiS_2_/C and NiS_2_/C@CNF were assessed using an X-ray diffraction (XRD) analysis and are illustrated in Figure 3a. The diffraction peaks at 27.1°, 31.5°, 35.3°, 38.7°, 45.1°, and 53.5° are assigned to the (111), (200), (210), (211), (220), and (311) crystallographic planes of cubic-phase NiS_2_ (JCPDS: 65-3325), respectively [35]. The sharp peaks observable in both samples indicate well-defined crystallinity. Figure 3b presents the XPS survey spectrum of the NiS_2_/C@CNF, revealing the elemental nature of the S, N, C, O and Ni in the NiS_2_/C@CNF sample. Figure 3c implies the high-resolution XPS spectrum of C, where the two peaks at 283.7 eV and 285.3 eV are assigned to C-S and C-N bonds [36], confirming the doping of S and N into the carbon. In Figure 3d, the high-resolution XPS spectrum of Ni displays peaks at 880.4 eV and 863.3 eV, associated with the Ni^3+^ 2p_1/2_ and Ni^3+^ 2p_3/2_, and peaks at 875.2 eV and 856.4 eV corresponding to the Ni^2+^ 2p_1/2_ and Ni^2+^ 2p_3/2_ [37]. The high-resolution XPS spectrum of sulfur (Figure 3e) features a peak at 169.1 eV, indicative of S–C bonding, further demonstrating the incorporation of S into the carbon structure. Additionally, the peaks at 168.2 eV, 164.3 eV, and 163.4 eV are related to the S 2p_1/2_, S 2p_3/2_, and Ni–S bonds, while an S–S bond is identified at 162.4 eV, providing evidence for the successful synthesis of NiS_2_ [38]. The content of NiS_2_ was calculated by TGA. Based on the mass loss observed in Figure 3f, the content of NiS_2_ within the NiS_2_/C@CNF was calculated to be approximately 73%.

**Figure 4:**
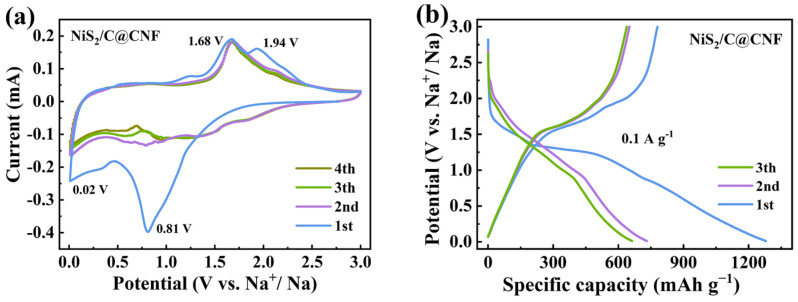
(**a**) Cyclic voltammetry of NiS_2_/C@CNF electrode at 0.2 mV s^−1^. (**b**) Charge–discharge curves of NiS_2_/C@CNF electrode at 0.1 A g^−1^.

Figure 4a depicts the CV curves of the NiS_2_/C@CNF at 0.2 mV s^−1^. A broad reduction peak is observed at 0.81 V, which is indicative of the insertion of Na into the NiS_2_/C@CNF (NiS_2_ + xNa^+^ + xe^−^ → Na_x_NiS_2_) and the production of an SEI layer [23]. A smaller reduction peak at 0.02 V is identified as Na intercalation into carbon. Additionally, the oxidation peaks at 1.68 V and 1.94 V are attributed to the extraction of Na from the NiS_2_/C@CNF (Na_x_NiS_2_ + (4−x)Na + (4−x)e^−^ → 2Na_2_S + Ni) [35]. The oxidation peak at 1.94 V disappears from the following cycles, unraveling the irreversible extraction of sodium ions. The subsequent cycle curves almost completely overlap, suggesting the exceptional reversibility of the NiS_2_/C@CNF. Additionally, the peak close to 0.75 V is indicative of the reversible Na^+^ intercalation into the NiS_2_/C@CNF, while the peak at 1.68 V denotes the deintercalation of Na^+^ [25]. Figure 4b exhibits the galvanostatic charge–discharge (GCD) profiles of the NiS_2_/C@CNF electrode, demonstrating initial charge/discharge capacities of 1279.1/780.5 mA h g^−1^ at 0.1 A g^−1^, respectively, delivering an initial coulomb efficiency (ICE) of 61%. The observed capacity decay is because of the generation of SEI and other irreversible processes [26].

**Figure 5:**
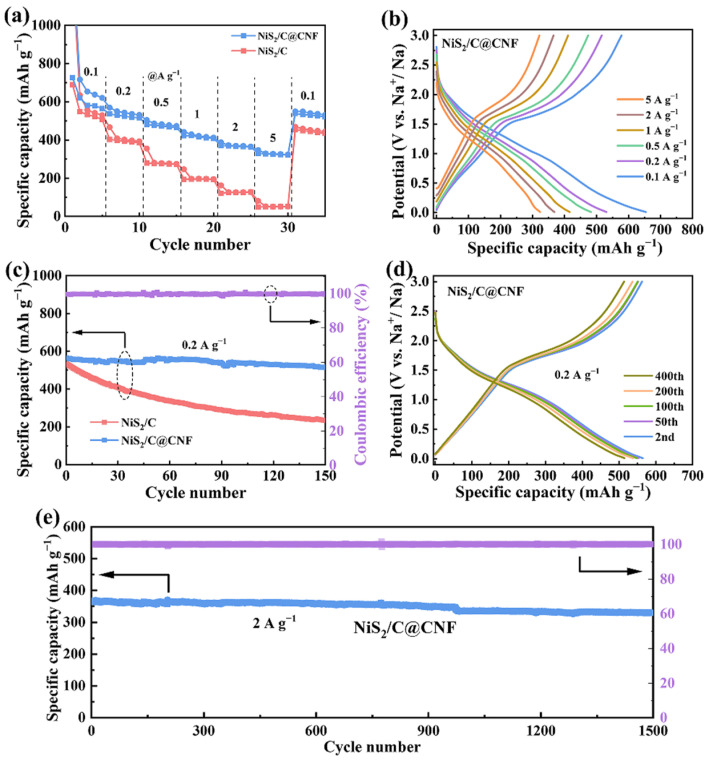
(**a**) The rate capability of the NiS_2_/C and NiS_2_/C@CNF and (**b**) the GCD profiles of the NiS_2_/C@CNF. (**c**) The cycle capability of the two electrodes at 0.2 A g^−1^ and (**d**) the GCD profiles of the NiS_2_/C@CNF. (**e**) The extended cycle capability of the NiS_2_/C@CNF electrode at 2 A g^−1^.

Figure 5a compares the rate properties of the NiS_2_/C and NiS_2_/C@CNF electrodes and Figure 5b displays the GCD curves of the NiS_2_/C@CNF electrode. As the current density increases from 0.1 to 5 A g^−1^, the reversible capacities of the NiS_2_/C@CNF electrode are 579.3, 516.1, 473.4, 410.9, 365.5, and 321.3 mA h g^−1^. The capacity at 5 A g^−1^ is 55.4% of that at 0.1 A g^−1^, signifying advanced rate behavior indicative of robust reaction kinetics (surpassing that of many NiS_2_-based electrodes, Figure S2). In contrast, the NiS_2_/C sample provides a reversible capacity of 532.3, 389.1, 273.7, 194.9, and 124.7 mA h g^−1^ at 0.1 to 2 A g^−1^, and the capacity dramatically declines to 51.3 mA h g^−1^ at 5 A g^−1^ and becomes nearly negligible, underscoring its limited rate properties. The enhanced rate behavior of the NiS_2_/C@CNF is related to its distinctive structure, in which the NS-CNS and carbon nanofibers form a 3D carbon architecture. This structure provides efficient electron transport pathways and promotes the homogeneous distribution of electroactive sites. Furthermore, the encapsulation of NiS_2_ nanoparticles within the conductive carbon architecture markedly boosts the conductivity and durability of the structure of the NiS_2_/C@CNF electrode. Figure 5c presents the cycling stability of the NiS_2_/C and NiS_2_/C@CNF at 0.2 A g^−1^. The NiS_2_/C@CNF electrode exhibits prominent cycling robustness, with a capacity retention of 513.8 mA h g^−1^ after 150 cycles, maintaining a capacity of 91.3%. The NS-CNS demonstrates a reversible specific capacity of 265.7 mA h g^−1^ (Figure S3). In contrast, the NiS_2_/C suffers from a rapid capacity decrease, only retaining 233 mA h g^−1^ after 150 cycles, implying 44.8% capacity retention. This significant difference underscores the distinctive structure of the NiS_2_/C@CNF electrode. Figure 5d provides the GCD curves of the NiS_2_/C@CNF electrode, which exhibit a high degree of overlap throughout the entire cycling process and underscore the excellent cycling stability. Figure 5e extends the investigation to the long-term cycling performance at 2 A g^−1^. After 1500 cycles, the NiS_2_/C@CNF electrode retains a capacity of 329.9 mA h g^−1^, with an impressive retention rate of 89.9%, showcasing exceptional long-term durability. According to the above findings, it is clear that the carbon nanofibers competently prevent the clustering of the NiS_2_ nanoparticles, which promotes the exposure of active sites, enabling the high reversible capacity of the NiS_2_/C@CNF electrode. Additionally, the 3D framework structure provides sufficient buffering space to accommodate the volumetric changes in the NiS_2_ nanoparticles, which is vital for maintaining the structural durability and cycling performance of the NiS_2_/C@CNF electrode.

**Figure 6:**
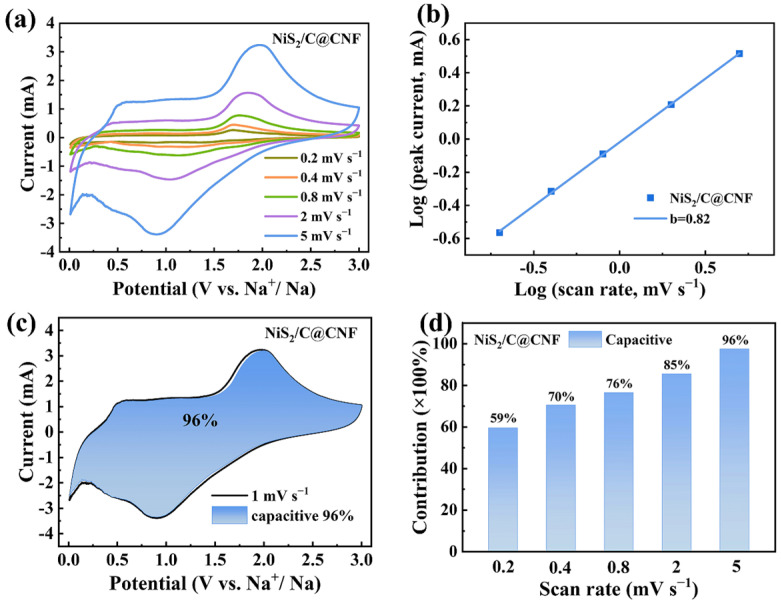
(**a**) CV curves of NiS_2_/C@CNF electrode. (**b**) B-values. (**c**,**d**) Capacitive contribution of NiS_2_/C@CNF electrode.

To elucidate the reaction kinetics of the NiS_2_/C@CNF, cyclic voltammetry (CV) tests were employed. As displayed in Figure 6a, the CV curves of the NiS_2_/C@CNF electrode maintain a consistent shape across the different scan rates, though slight shifts in the oxidation and reduction peaks are observed, attributed to polarization effects [39]. This stability in shape suggests an efficient charge transfer and robust structural integrity during the electrochemical process. The correlation between the peak current (i) and scan rate (v) can be obtained by the following equation: *i* = *a*^v^*b* [40]. A *b*-value of 0.5 indicates that the charge storage process is predominantly diffusion-controlled, whereas a *b*-value of 1 signifies that the current response is primarily governed by capacitive behavior [41]. The *b*-value of the NiS_2_/C@CNF electrode is 0.82 (Figure 6b), proving that the sodium-ion storage mechanism is predominantly governed by capacitive behavior. This suggests that the charge storage process involves rapid surface reactions, contributing to the high-rate performance. Pseudocapacitive sodium storage behavior involves surface-confined reactions, which facilitate rapid charge transfer processes and minimize structural degradation during cycling. The pseudocapacitive contribution is quantified using the formula *i* = *k*_1_v + *k*_2_v^1/2^ [42,43], where *k*_1_v reflects the surface-dominated sodium storage mechanism, while *k*_2_v^1/2^ represents the diffusion-controlled energy storage mechanism. As depicted in Figure 6c,d, the NiS_2_/C@CNF electrode exhibits pseudocapacitive contributions of 59%, 70%, 76%, 85%, and 96% from 0.2 mV s^−1^ to 5 mV s^−1^, respectively. The progressively higher pseudocapacitive contributions at increased scan rates underscore the dominant surface-driven charge storage mechanism, which effectively strengthens the reaction kinetics and overall performance of the NiS_2_/C@CNF electrode [44].

**Figure 7:**
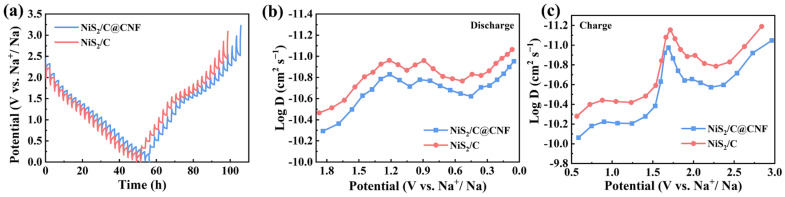
(**a**) GITT profiles and (**b**,**c**) D_Na_^+^ of NiS_2_/C and NiS_2_/C@CNF electrodes.

To investigate in-depth the reaction dynamics of the NiS_2_/C and NiS_2_/C@CNF electrodes, GITT tests were conducted (Figure 7a). The sodium-ion diffusion coefficient (D_Na_^+^) was determined using the following equation [45,46]:

D = 4*l* (ΔE_s_/ΔE_τ_)^2^/πτ (1)

where *l* stand for the sodium ions’ diffusion length (cm), ΔE_S_ represents the steady-state voltage change, ΔE_*τ*_ symbolizes the transient voltage change, and τ is the duration of the applied current pulse. The calculated D_Na_^+^ values for both electrodes during the discharge and charge cycles are presented in Figure 7b,c, ranging from 10^−10^ to 10^−11^ cm^2^ s^−1^. Notably, the D_Na_^+^ values of the NiS_2_/C@CNF consistently surpass those of the NiS_2_/C electrode during the entire process, indicative of the superior fast-rate behavior of the NiS_2_/C@CNF electrode. This observation demonstrates the enhanced reaction dynamics of the NiS_2_/C@CNF electrode, attributable to its unique 3D carbon backbone, which facilitates more efficient ion transport and improved conductivity. Its superior diffusion characteristics underscore the potential of the NiS_2_/C@CNF architecture for advanced sodium-ion storage applications.

## 3. Experimental Section

### 3.1. Synthesis of the Carbon Nanofibers Film (CNF)

To synthesize the carbon nanofibers (CNFs), 1 g of polyacrylonitrile (PAN) was dissolved in 10 mL of dimethylformamide (DMF) under continuous magnetic stirring for 12 h. The resulting electrospinning precursor was then transferred into a plastic syringe. The aluminum foil collector was positioned approximately 16 cm from the syringe needle, and a voltage of 15 kV was applied to the collector. After stabilization in air, the precursor film underwent annealing at 800 °C for 2 h in a N_2_ atmosphere, leading to the fabrication of the carbon nanofiber film.

### 3.2. Fabrication of Ni(OH)_2_/N-polysaccharide@CNFs

Using a hydrothermal process, 0.6 g of glucose (C_6_H_12_O_6_), 0.4 g of NiSO_4_·6H_2_O, and 0.4 g of hexamethylenetetramine (C_6_H_12_N_4_) were dissolved in 35 mL of water to form a uniform solution, which was positioned in a Teflon-lined autoclave. A piece of CNFs film was subsequently immersed in the prepared solution. The reaction mixture was subjected to 180 °C for 10 h; the film was washed with deionized water and dried, yielding the Ni(OH)_2_/N-polysaccharide@CNFs composite.

### 3.3. Synthesis of the NiS_2_/C@CNF

During the fabrication of the NiS_2_/C@CNF, the Ni(OH)_2_/N-polysaccharide@CNFs composite and sublimed sulfur were positioned at the downstream and upstream sections of the furnace chamber, respectively. They were then heated to 550 °C for 2 h in a N_2_ atmosphere; the Ni(OH)_2_ transformed into NiS_2_, and the N-polysaccharide transformed into nitrogen and sulfur co-doped carbon nanosheets, leading to the generation of the NiS_2_/C@CNF composite. As a comparison, 0.6 g of C_6_H_12_O_6_, 0.4 g of NiSO_4_·6H_2_O, and 0.4 g of C_6_H_12_N_4_ underwent a hydrothermal reaction under the same conditions without the addition of CNF to obtain Ni(OH)_2_/N-polysaccharide. Subsequently, the same vapor-phase sulfidation process was applied, resulting in NiS_2_/C.

### 3.4. Materials Characterization

The crystal structure and chemical composition of the samples were comprehensively characterized using a suite of advanced techniques, including X-ray diffraction (XRD, Rigaku SmartLab SE diffractometer, Cu Kα radiation, Rigaku, Tokyo, Japan), X-ray photoelectron spectroscopy (XPS, Thermo Scientific, Waltham, MA, USA), Raman spectroscopy (Horiba LabRAM HR Evolution system, Horiba, Kyoto, Japan). Additionally, the surface morphology and microstructure were examined through field-emission scanning electron microscopy (FE-SEM, JSM-7900F, Hitachi, Tokyo, Japan) and transmission electron microscopy (TEM, JEOL JEM-F200 microscope, JEOL, Tokyo, Japan).

### 3.5. Electrochemical Characterizations

All the electrochemical evaluations were performed using CR2032-type coin cells. For the NiS_2_/C electrode, the NiS_2_/C composite was blended with Super P and polyvinylidene fluoride (PVDF) at a weight ratio of 8:1:1 to create a slurry in N-methyl-2-pyrrolidone (NMP). This slurry was then uniformly coated onto copper foil and dried in a vacuum oven at 100 °C for 12 h. For the NiS_2_/C@CNF electrode, the NiS_2_/C@CNF film was cut into 14 mm diameter discs to function as a self-supporting electrode. The electrolyte solution consisted of 1 M NaClO_4_ in propylene carbonate (PC), with a glass fiber membrane (Whatman, GF/D) as the separator and a sodium metal foil as the counter electrode. All the cells were assembled in an argon-filled glovebox. Galvanostatic charge–discharge measurements were conducted within a voltage range of 0.01–3.0 V versus Na/Na^+^ using a CT2001A cell testing system (LAND Electronic Co., Wuhan, China). Cyclic voltammetry (CV) was carried out using an electrochemical workstation (CH Instruments, Bee Cave, TX, USA, model 660C). The galvanostatic intermittent titration technique (GITT) was employed with a pulse current of 100 mA g^−1^ for 1 h, followed by a 2 h rest interval.

## 4. Conclusions

In summary, we present a 3D carbon backbone encapsulation approach to enhance the Na-ion storage efficiency of a NiS_2_/C@CNF anode. By utilizing a hydrothermal method followed by gas-phase sulfidation, NiS_2_ nanoparticles are encapsulated within a carbon backbone, forming a self-supported film electrode. The 3D carbon backbone structure facilitates electron transport, thereby enhancing the reaction kinetics of the NiS_2_/C@CNF. Furthermore, the 3D carbon backbone accommodates the volumetric changes in NiS_2_ during charge/discharge cycles, optimizing its cycling stability. Owing to this distinctive encapsulation approach, the NiS_2_/C@CNF electrode exhibits a superior rate capability (579.3 mA h g^−1^ at 0.1 A g^−1^ and 321.3 mA h g^−1^ at 5 A g^−1^) and exceptional cycle durability (capacity retention of 89.9% after 1500 cycles at 2 A g^−1^).

## Data Availability

The data presented in this study are available on request from the corresponding author. The data are not publicly available due to institutional restrictions.

## Supplementary Materials

The following supporting information can be downloaded at: https://www.mdpi.com/article/10.3390/molecules29245906/s1, Figure S1: The nitrogen adsorption–desorption isotherms of the NiS_2_/C@CNF sample. Figure S2: A comparison of the rate capability of the NiS_2_/C@CNF and other NiS_2_-based materials. Figure S3: The cycle capability of the NS-CNS, which is obtained by removing NiS_2_ from the NiS_2_/C@CNF. References [23,26,47] are cited in the Supplementary Materials.
