# Supplementary material for: A 3D Carbon Architecture Encapsulation Strategy for Boosting the Performance of Nickel Disulfide as an Anode for Sodium-Ion Batteries
Source: Molecules. 2024 Dec 14;29(24):5906. doi: 10.3390/molecules29245906 (PMC11677060; doi:10.3390/molecules29245906)
Supplement: Supplementary file 1 [file molecules-29-05906-s001.zip › molecules-3357206-supplementary.pdf]

A 3D carbon architecture encapsulation strategy in boosting the performance of nickel disulfide as an anode for sodium-ion batteries

Yuzhu Li [a, *], Yuanfei Gao [a,*], Mengyuan Zhang [a], Boying Zhang [a], Bingke Li [b],*

[a] College of Chemistry and Pharmaceutical Engineering, Nanyang Normal University, Nanyang 473061, P. R. China.

[b] School of Biological and Chemical Engineering, Nanyang Institute of Technology, Nanyang 473004, P. R. China.

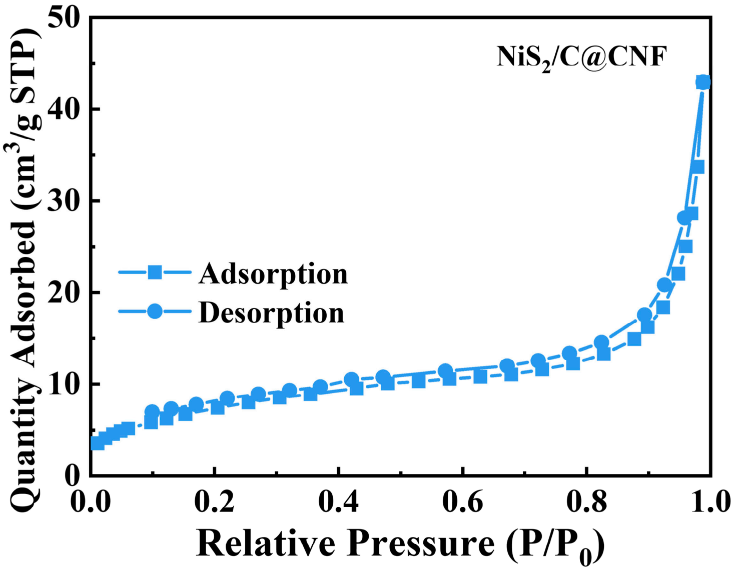

Figure S1 Nitrogen adsorption–desorption isotherms of NiS_2/C@CNF sample.

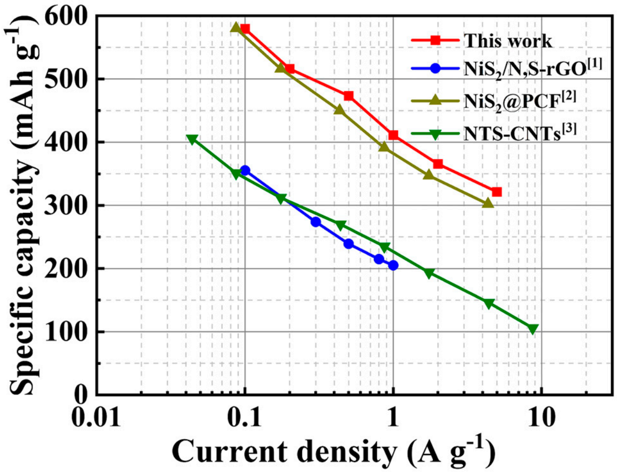

Figure S2 Comparison of the rate capability of NiS_2/C@CNF and other NiS_2-based materials.[1-3]

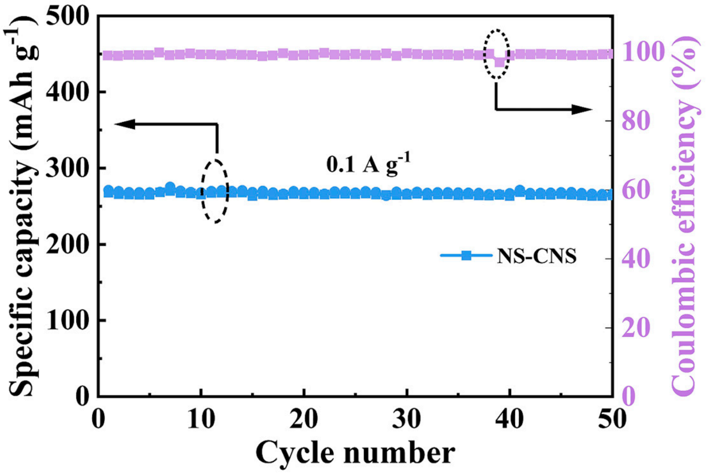

Figure S3 The cycle capability of the NS-CNS, which is obtained by removing NiS_2 from NiS_2/C@CNF.

References

1. Dong, X.; Chen, F.; Chen, G.; Wang, B.; Tian, X.; Yan, X.; Yin, Y.-X.; Deng, C.; Wang, D.; Mao, J., *et al.*, NiS_2 nanodots on N,S-doped graphene synthesized via interlayer confinement for enhanced lithium-/sodium-ion storage, J. Colloid Interf. Sci. **2022**, 619, 359-368.

2. Chen, Q.; Sun, S.; Zhai, T.; Yang, M.; Zhao, X., and Xia, H., Yolk – shell NiS_2 nanoparticle–embedded carbon fibers for flexible fiber–shaped sodium battery, Adv. Energy Mater. **2018**, 8, 1800054.

3. Sadaqat, A.; Ali, G.; ul Hassan, M.; Iftikhar, F.J., and Abbas, S., Ni_3S_4/SnS/Graphene Oxide/Carbon Nanotube Composites as Anodes for Na-Ion Batteries, ACS Appl. Nano Mater. **2023**, 6, 1996-2008.
